# The transcription factor SlHY5 regulates the ripening of tomato fruit at both the transcriptional and translational levels

**DOI:** 10.1038/s41438-021-00523-0

**Published:** 2021-04-01

**Authors:** Weihao Wang, Peiwen Wang, Xiaojing Li, Yuying Wang, Shiping Tian, Guozheng Qin

**Affiliations:** 1 Key Laboratory of Plant Resources, Institute of Botany, the Innovative Academy of Seed Design, Chinese Academy of Sciences, No. 20 Nanxincun, Xiangshan, Haidian District, 100093 Beijing, China; 2 University of Chinese Academy of Sciences, 100049 Beijing, China

**Keywords:** Plant physiology, Plant molecular biology

## Abstract

Light plays a critical role in plant growth and development, but the mechanisms through which light regulates fruit ripening and nutritional quality in horticultural crops remain largely unknown. Here, we found that ELONGATED HYPOCOTYL 5 (HY5), a master regulator in the light signaling pathway, is required for normal fruit ripening in tomato (*Solanum lycopersicum*). Loss of function of tomato *HY5* (*SlHY5*) impairs pigment accumulation and ethylene biosynthesis. Transcriptome profiling identified 2948 differentially expressed genes, which included 1424 downregulated and 1524 upregulated genes, in the *Slhy5* mutants. In addition, genes involved in carotenoid and anthocyanin biosynthesis and ethylene signaling were revealed as direct targets of SlHY5 by chromatin immunoprecipitation. Surprisingly, the expression of a large proportion of genes encoding ribosomal proteins was downregulated in the *Slhy5* mutants, and this downregulation pattern was accompanied by a decrease in the abundance of ribosomal proteins. Further analysis demonstrated that SlHY5 affected the translation efficiency of numerous ripening-related genes. These data indicate that SlHY5 regulates fruit ripening both at the transcriptional level by targeting specific molecular pathways and at the translational level by affecting the protein translation machinery. Our findings unravel the regulatory mechanisms of SlHY5 in controlling fruit ripening and nutritional quality and uncover the multifaceted regulation of gene expression by transcription factors.

## Introduction

Light not only serves as a source of energy for plant photosynthesis, but also represents an important cue that regulates plant growth and development, including seed germination^1^, seedling growth^2^, flowering^3^, and eventually senescence^4^. To sense light, plants have evolved several classes of photoreceptors, among which cryptochromes and phototropins monitor blue light, phytochromes monitor red/far-red light, and UVR8 monitors UV-B light^5^. Upon light perception, the photoreceptors interpret and transduce light signals to the downstream core signaling networks, which leads to remodeling of the transcriptome and changes in growth and development^6^.

Transcriptional regulation plays critical roles in light-regulated growth and developmental processes. Nearly 20–35% of plant genes display massive transcriptional reprogramming under light^7^. Several transcription factors, such as PHYTOCHROME-INTERACTING FACTORs (PIFs) and ELONGATED HYPOCOTYL 5 (HY5), which exhibit antagonistic actions, have been identified as the key components in the light signaling pathway^8^. PIFs belong to the family of basic helix-loop-helix transcription factors, whereas HY5 is a member of the basic leucine zipper (bZIP) transcription factor family^9,10^. The stability of both PIFs and HY5 is regulated by CONSTITUTIVE PHOTOMORPHOGENIC 1 (COP1), an E3 ubiquitin ligase that mediates protein ubiquitination and degradation by the 26S proteasome^11^. COP1 directly targets HY5 for degradation but positively affects the PIF protein levels via an indirect effect^12^. In the presence of light, COP1 is repressed by photoreceptor phytochromes and cryptochromes, which results in instable PIFs and the accumulation of HY5 and thereby in the promotion of photomorphogenesis^13^.

As a central positive regulator in the light signaling pathway, HY5 is involved in fundamental developmental processes in plants, including cell proliferation, cell elongation, and chloroplast development^14^. Interestingly, in tomato (*Solanum lycopersicum*) fruit, HY5 regulates the accumulation of carotenoids^15^, a group of 40-carbon isoprenoid compounds that contribute to fruit color and nutritional quality in various horticultural crops. HY5 might act in concert with other components in the light signaling pathway, including PIF1a^16^, DAMAGED DNA BINDING PROTEIN 1/HIGH PIGMENT 1 (DDB1/HP1)^17^, DEETIOLATED 1/HIGH PIGMENT 2 (DET1/HP2)^18^, CULLIN 4 (CUL4)^17^, and COP1-like^15^, to modulate fruit carotenoid accumulation in tomato. However, the direct targets modulated by HY5 in horticultural crops and the mechanisms underlying its transcriptional regulation remain largely unknown. Furthermore, whether HY5 modulates other physiological aspects of fruit development remains unclear. Such information could be highly useful for unraveling the regulatory cascade controlled by HY5 and for understanding the connections with other regulatory networks that control fruit quality.

In the present study, to characterize the biological function of tomato HY5 (SlHY5) and its regulatory mechanisms, we generated *Slhy5* mutants using CRISPR/Cas9 genome editing technology. We found that the *SlHY5* mutation impaired the normal fruit ripening process through reduced pigment accumulation and ethylene release. A transcriptome analysis coupled with chromatin immunoprecipitation (ChIP) indicated that SlHY5 directly bound to promoters of genes involved in carotenoid and anthocyanin biosynthesis, and genes associated with ethylene generation. Surprisingly, we found that SlHY5 influenced the protein translation efficiency of numerous ripening genes by targeting ribosomal protein genes. Our findings suggest that SlHY5 regulates fruit ripening both at the transcriptional level by targeting specific molecular pathways and at the translational level by affecting the protein translation machinery.

## Results

### CRISPR/Cas9-engineered mutations in *SlHY5* cause abnormal ripening in tomato fruit

**Fig. 1:**
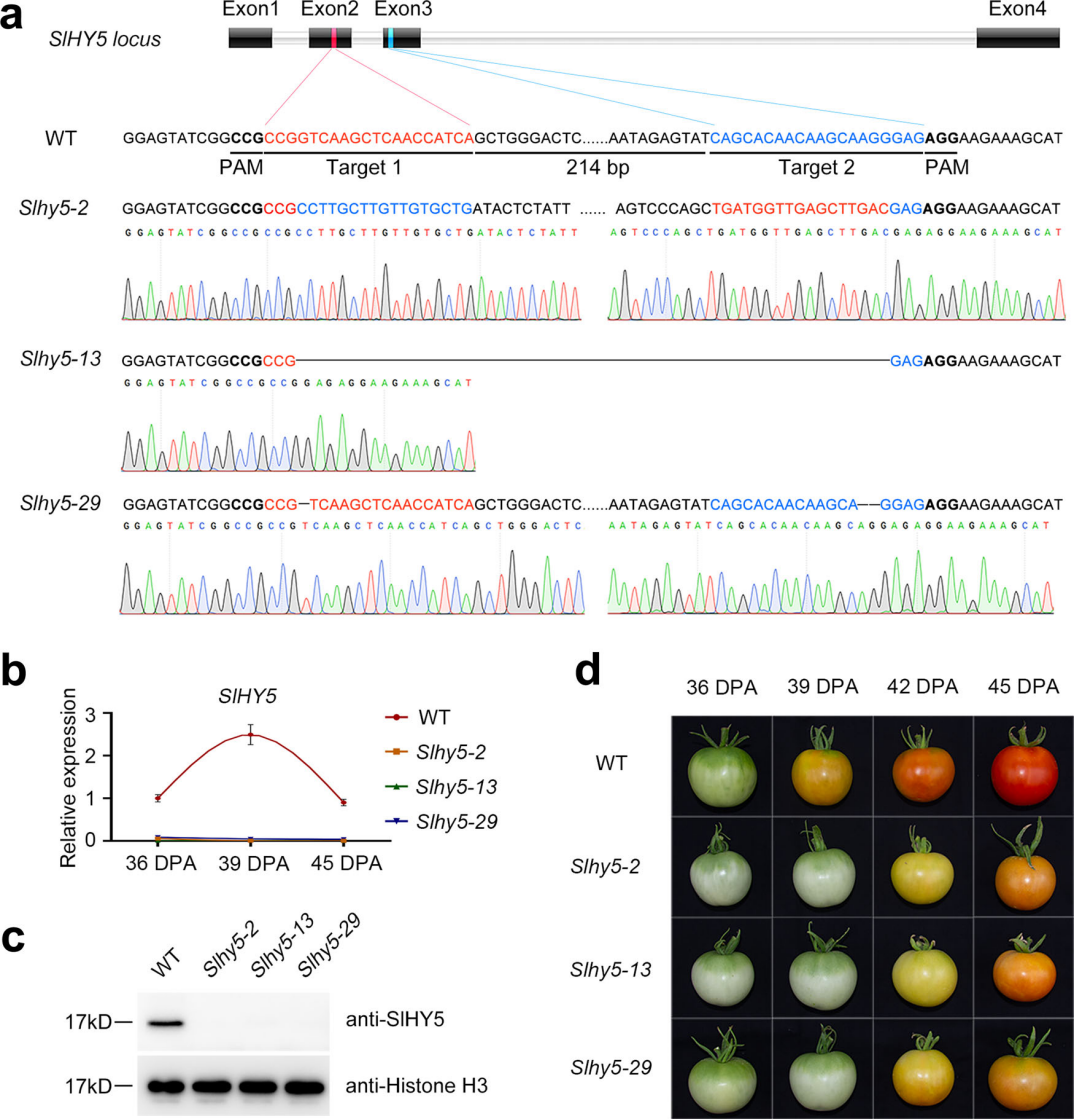
SlHY5 is required for the normal ripening of tomato fruit. **a** Genotype of mutations in the *SlHY5* locus generated by the CRISPR/Cas9 genome editing system. Two target sequences were designed to specifically target the second and third exons with a 214-bp interval. The red and blue letters indicate the sequences of targets 1 and 2, respectively. The protospacer adjacent motif (PAM) is indicated. The mutations in the transgenic plants were confirmed by sequencing the genomic regions flanking the target sites. The sequences of the wild-type (WT) plants and three homozygous mutant lines (*Slhy5-2*, *Slhy5-13*, and *Slhy5-29*) are shown. **b** Transcript levels of *SlHY5* in wild-type and *Slhy5* mutant fruits determined by quantitative real-time PCR. **c** SlHY5 protein levels in wild-type and *Slhy5* mutant fruits determined by western blotting. The histone H3 protein levels were used as a loading control. **d** Ripening phenotype of *Slhy5* mutants. Wild-type and *Slhy5* mutant fruits at 36, 39, 42, and 45 days post anthesis (DPA) are shown

In recent years, CRISPR/Cas9 gene-editing technology has been widely used in studies of plant gene function. To better understand the biological function of *SlHY5* (*Solyc08g061130*), the only *HY5* gene in tomato, we generate mutations in the *SlHY5* locus using the CRISPR/Cas9 gene-editing system. Two specific target sites were designed in the second and third exons (Fig. 1a). We ultimately obtained 31 independent transgenic lines of the T_0_ generation through *Agrobacterium tumefaciens*-mediated transformation and the mutation of *SlHY5* was verified by sequencing genomic regions flanking the target sites. We isolated three distinct homozygous mutant lines (*Slhy5-2*, *Slhy5-13*, and *Slhy5-29*) of the T_1_ generation and used these for further analysis. A large 248-bp inversion between the two targets was found at the *SlHY5* locus in the *Slhy5-2* line, whereas a deletion of 248 bp appeared in the *Slhy5-13* line, and the *Slhy5-29* line harbored a 1-bp deletion in the first target and a 2-bp deletion in the second target (Fig. 1a). The three mutants were predicted to produce nonfunctional truncated SlHY5 proteins. We performed an off-target analysis and found that no genome editing occurred at the most likely off-target sites, which were predicted using CRISPR-P, in the three mutagenesis lines (Supplementary Fig. 1). Quantitative real-time PCR and western blotting analyses were performed to determine the expression of SlHY5 in the *Slhy5* mutants at both the mRNA and protein levels. The *SlHY5* transcript was almost completely silent in the *Slhy5* mutants, and the SlHY5 protein was also completely undetectable (Fig. 1b, c). These results confirmed that *SlHY5* was successfully mutated.

The fruit ripening progress was markedly delayed in the *Slhy5-2*, *Slhy5-13*, and *Slhy5-29* mutant lines compared with the wild-type plants (Fig. 1d). The onset of ripening in the wild-type fruit occurred at 39 days post anthesis (DPA), whereas a visible color change started to be observed in the fruit of *Slhy5* mutants at 42 DPA. At 45 DPA, the wild-type fruit was fully ripe and showed a red color, whereas the fruits of the *Slhy5* mutants remained orange. Even at the final stage of fruit ripening, the fruits of the *Slhy5* mutants could not turn completely red and showed a light-red color (Supplementary Fig. 2). In addition, the green fruits of the *Slhy5* mutants were whiter than those of the wild-type plants. The phenotype of delayed fruit ripening was also observed in the T_2_ generation, which further confirmed that *SlHY5* regulates the ripening of tomato.

Seedlings of the *Slhy5* mutants growing under standard conditions displayed a significantly increased hypocotyl growth compared with the wild-type seedlings. After the transplantation of 4-week-old seedlings into the field, the *Slhy5* mutants exhibited a higher mortality rate than the wild-type plants. Moreover, the *Slhy5* mutant plants showed pale green leaves and pale yellow flowers (data not shown).

### The *Slhy5* mutants exhibit impaired expression of ripening-related genes

**Fig. 2:**
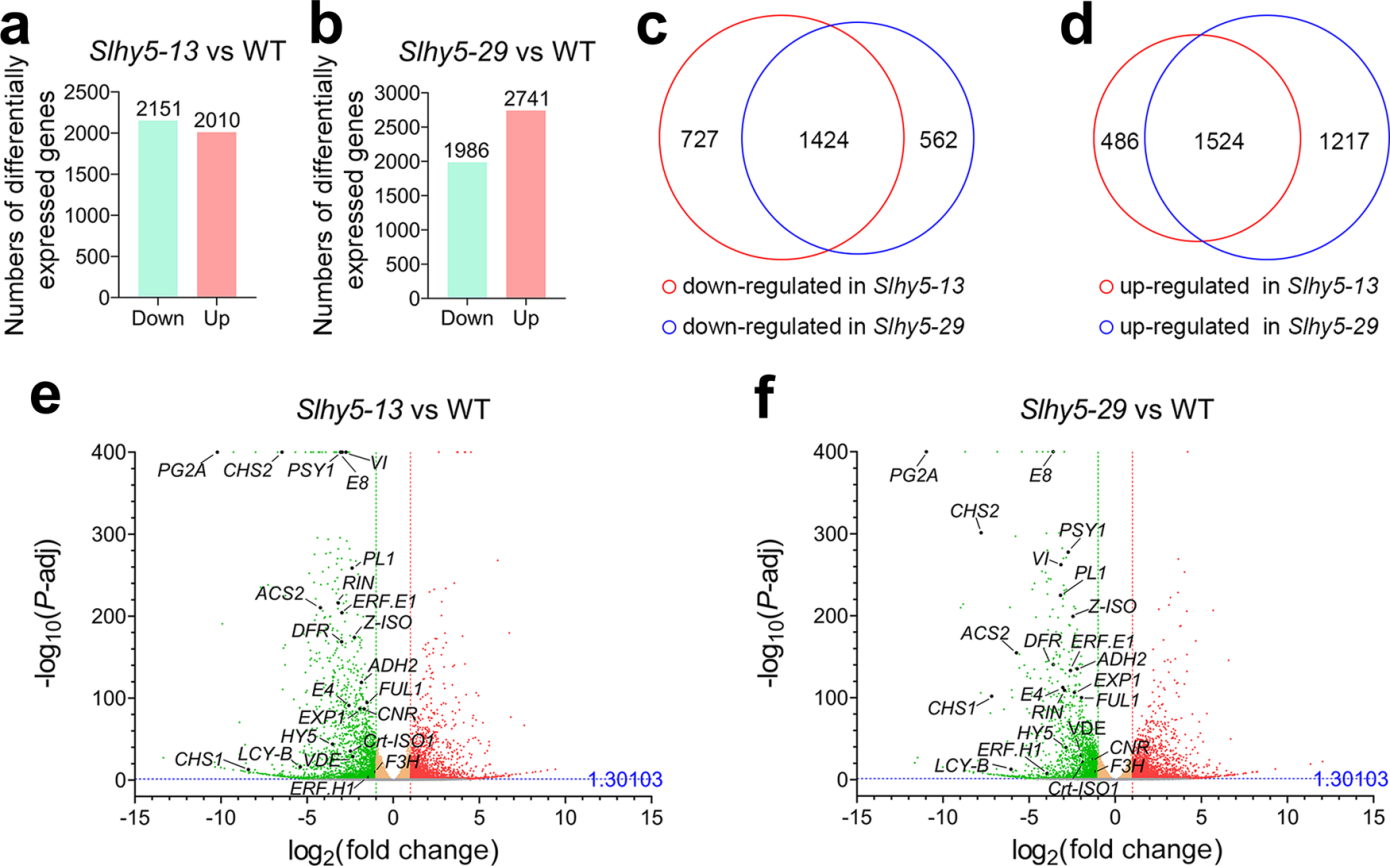
Expression of numerous ripening-related genes is decreased in the *Slhy5* mutants. **a**, **b** Number of differentially expressed genes that were downregulated or upregulated in the *Slhy5-13* (**a**) and *Slhy5-29* (**b**) mutants versus wild-type (WT) plants at 39 days post anthesis (DPA). **c**, **d** Venn diagrams showing the overlapping downregulated (**c**) and upregulated (**d**) genes that were differentially expressed in the *Slhy5-13* and *Slhy5-29* mutants. **e**, **f** Volcano diagrams revealing the differentially expressed genes in the *Slhy5-13* (**e**) and *Slhy5-29* (**f**) mutant versus WT plants. The colored spots indicate *P*-adj < 0.05, which indicates that the differences in gene expression are significant. The green and red spots show downregulated and upregulated genes with more than twofold changes, respectively. Some of the ripening-related genes are marked by black spots. *ACS2* *1-aminocyclopropane-1-carboxylic acid synthase 2*, *ADH2* a*lcohol dehydrogenase 2*, *ANS* *anthocyanidin synthase*, *CHI* c*halcone-flavonone isomerase*, *CHS1* *chalcone synthase 1*, *CHS2* *chalcone synthase 2*, *CNR* *colorless non-ripening*, *Crt-ISO1* c*arotene isomerase 1*, *DFR* *dihydroflavonol-4-reductase*, *E4* *ethylene response gene 4*, *E8* *ethylene response gene 8*, *ERF. E1* *ethylene response factor E1*, *ERF. H1* *ethylene response factor H1*, *EXP1* *expansin 1*, *F3H* *flavanone 3-hydroxylase*, *F3*′*H* *flavonoid 3*′*-hydroxylase*, *FUL1* *FRUITFULL 1*, *HY5* *ELONGATED HYPOCOTYL5*, *LCY-B* *lycopene beta-cyclase*, *PG2A* *polygalacturonase 2A*, *PL1* *pectate lyase 1*, *PSY1* *phytoene synthase 1*, *RIN* *ripening inhibitor*, *VDE* *violaxanthin de-epoxidase*, *VI* *vacuolar invertase*, *Z-ISO* *15-cis-zeta-carotene isomerase*

To determine how *SlHY5* affects tomato fruit ripening, we performed a comparative transcriptome analysis between the wild-type plants and the *Slhy5* mutants (*Slhy5-13* and *Slhy5-29*) at 39 DPA based on RNA sequencing (RNA-seq). In total, we obtained >492 million clean reads, and >95% of these reads could be mapped to unique loci in the reference genome. A bioinformatics analysis identified 4161 differentially expressed genes, which included 2151 downregulated [log_2_(fold change) < −1, *P-*adj < 0.05] and 2010 upregulated [log_2_(fold change) > 1, *P*-adj < 0.05] genes in the *Slhy5-13* line (Fig. 2a and Supplementary Table 1). Moreover, 1986 downregulated and 2741 upregulated genes were differentially expressed in the *Slhy5-29* line (Fig. 2b and Supplementary Table 2). Overall, 1424 downregulated and 1524 upregulated differentially expressed genes were found in both mutants (Fig. 2c, d and Supplementary Table 3). A high Pearson correlation coefficient was found between the two mutants, which represented the high similarity between them (Supplementary Fig. 3). Notably, the *SlHY5* mRNA level was significantly decreased in both lines (Fig. 2e, f and Supplementary Table 3), which suggested that genome editing might cause RNA instability due to frameshift mutations. Among the differentially expressed genes, genes involved in anthocyanin biosynthesis (*CHS1*, *CHS2*, *CHI*, *F3H*, *F3’H*, *DFR*, and *ANS*), ethylene biosynthesis and response (*ACS2*, *E4*, *E8*, *ERF.E1*, and *ERF.H1*), carotenoid metabolism (*PSY1*, *Z-ISO*, *Crt-ISO1*, *LCY-B*, and *VDE*), volatile aroma compound biosynthesis (*ADH2*), sucrose metabolism (*VI*), and cell wall degradation (*PG2A*, *PL1*, and *EXP1*) were substantially downregulated. The expression of some well-known ripening-related transcription factors, such as *RIN*, *CNR*, and *FUL1*, was also decreased in the *Slhy5* mutants (Fig. 2e, f).

### Carotenoid biosynthesis is inhibited in the *Slhy5* mutants

**Fig. 3:**
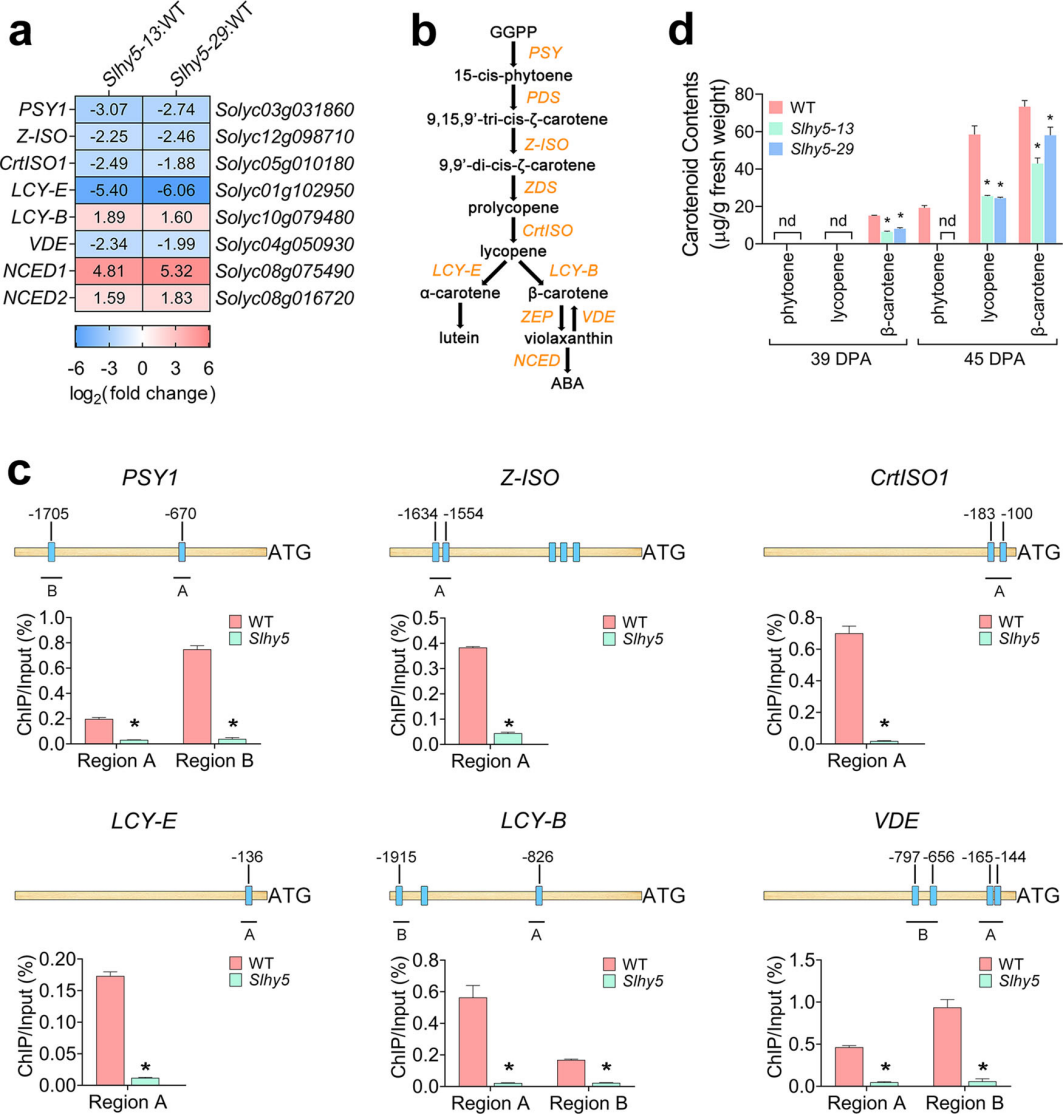
Carotenoid biosynthesis is regulated by SlHY5. **a** Heatmap showing the differentially expressed genes involved in carotenoid biosynthesis identified from the comparison of the *Slhy5* mutant (lines *Slhy5-13* and *Slhy5-29*) versus wild-type (WT) fruits at 39 days post anthesis (DPA) based on RNA-seq analysis. **b** Sketch diagram displaying the core carotenoid metabolism pathway. **c** ChIP-qPCR assays showing that SlHY5 binds to the promoters of genes involved in carotenoid biosynthesis. The promoter structures of the SlHY5 target genes are shown. The blue boxes represent ACGT-containing elements, and the numbers indicate the positions of these motifs relative to the translational start site. The black lines with uppercase letters represent the regions used for ChIP-qPCR. The values show the percentages of DNA fragments that coimmunoprecipitated with anti-SlHY5 antibodies relative to the input DNAs. *PSY1* *phytoene synthase 1*, *Z-ISO* *15-cis-zeta-carotene isomerase*, *Crt-ISO1* *carotene isomerase 1*, *LCY-E lycopene epsilon-cyclase*, *LCY-B* *lycopene beta-cyclase*, *VDE* *violaxanthin de-epoxidase*. **d** Carotenoid accumulation in *Slhy5* mutants at 39 and 45 DPA. The error bars represent the standard deviations from three independent experiments. The asterisks indicate significant differences (*P < 0.05)

The accumulation of carotenoids appears to be a typical feature during tomato fruit ripening. The RNA-seq assay revealed that eight genes (*PSY1*, *Z-ISO*, *Crt-ISO1*, *LCY-E*, *LCY-B*, *VDE*, *NCED1*, and *NCED2*) involved in carotenoid metabolism were differentially expressed in the *Slhy5* mutants (Fig. 3a). PSY is the rate-limiting enzyme in the carotenoid biosynthetic pathway (Fig. 3b). The tomato genome contains three *PSY* genes, and only *PSY1* is expressed specifically during fruit ripening and contributes to carotenoid accumulation in tomato fruit^19^. A ChIP-qPCR analysis was performed to identify whether genes (*PSY1*, *Z-ISO*, *Crt-ISO1*, *LCY-E*, *LCY-B*, and *VDE*) involved in the metabolism of lycopene, β-carotene, and phytoene, which are the main carotenoids in tomato fruit, were directly regulated by SlHY5.

We scanned the presence of ACGT-containing elements, which have been elucidated as the binding sites of HY5 (refs. ^20,21^), in the promoter regions of the candidate genes and found one to five ACGT-containing elements in the 2-kb region upstream from the start codon (ATG). For the ChIP-qPCR assay, cross-linked DNA–protein complexes were immunoprecipitated using affinity-purified anti-SlHY5 polyclonal antibodies. Specific primers were designed to amplify the promoter regions surrounding the ACGT-containing elements from the immunoprecipitated DNA. The binding of SlHY5 to the promoters was expressed as the relative amount of immunoprecipitated DNA fragments versus the input DNA fragments. As shown in Fig. 3c, SlHY5 bound to the promoters of all the candidate genes involved in carotenoid biosynthesis, which indicated that SlHY5 directly regulates these genes.

We next determined the contents of lycopene, β-carotene, and phytoene in tomato fruit (Fig. 3d). At 39 DPA, only β-carotene could be detected, and higher levels of carotenoid were found in the wild-type fruits than in the *Slhy5* mutants. At 45 DPA, phytoene was not detected in the *Slhy5* mutants but appeared in the wild-type fruits. Although lycopene and β-carotene could be detected in the *Slhy5* mutants at 45 DPA, their contents were significantly lower in the mutants than in the wild-type fruits. Taken together, these results indicated that SlHY5 affects carotenoid accumulation by targeting genes involved in the carotenoid biosynthetic pathway.

### Anthocyanin accumulation is suppressed in the *Slhy5* mutants

**Fig. 4:**
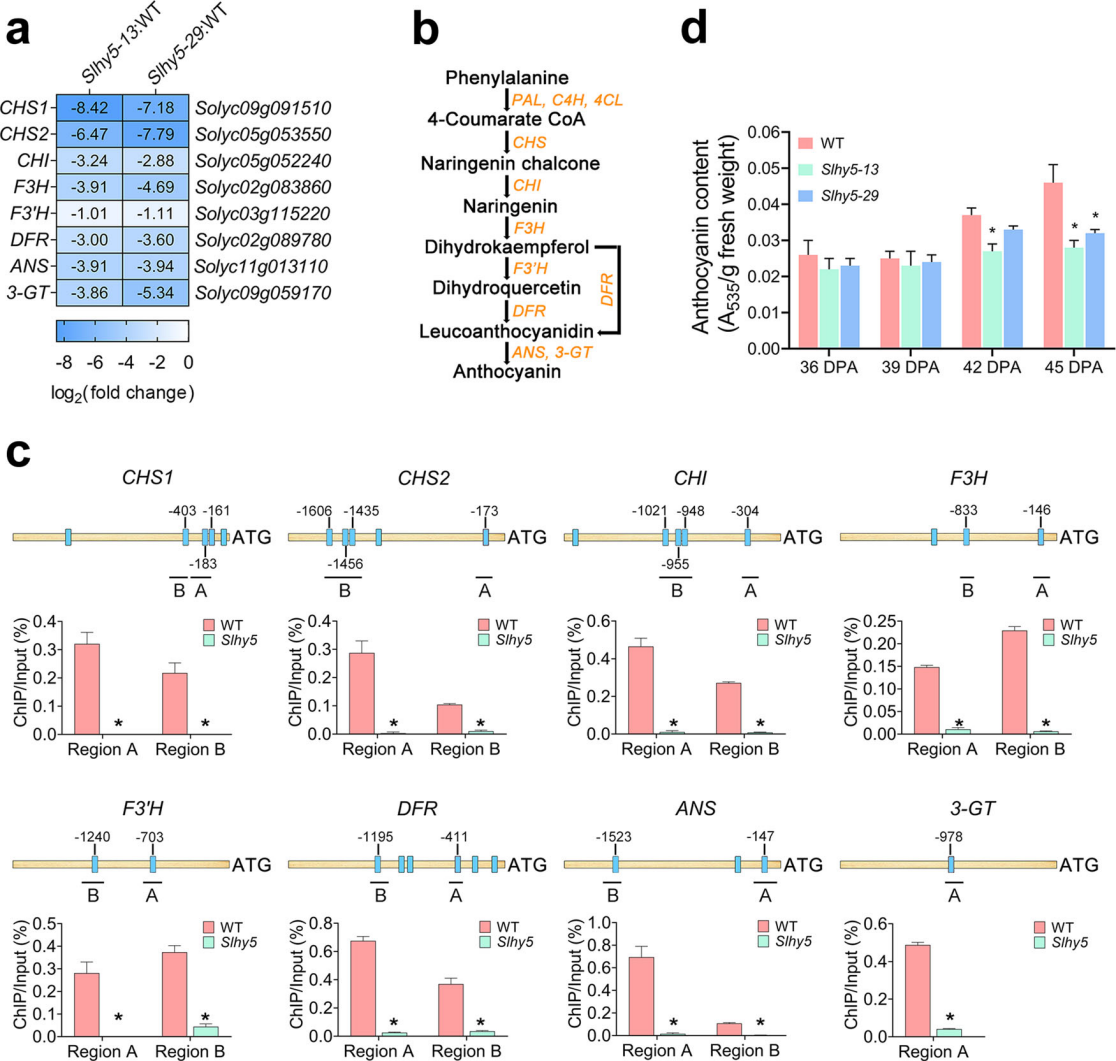
Anthocyanin biosynthesis is regulated by SlHY5. **a** Heatmap showing the differentially expressed genes involved in anthocyanin synthesis that were identified from the comparison of the *Slhy5* mutant (lines *Slhy5-13* and *Slhy5-29*) versus wild-type (WT) fruits at 39 days post anthesis (DPA) based on RNA-seq analysis. **b** Sketch diagram exhibiting the core anthocyanin biosynthesis pathway. **c** ChIP-qPCR assays showing that SlHY5 binds to the promoters of genes involved in anthocyanin synthesis. The promoter structures of the SlHY5 target genes are shown. The blue boxes represent ACGT-containing elements, and the numbers indicate the positions of these motifs relative to the translational start site. The black lines with uppercase letters represent the regions used for ChIP-qPCR. The values are the percentages of DNA fragments that coimmunoprecipitated with anti-SlHY5 antibodies relative to the input DNAs. *ANS* *anthocyanidin synthase*, *CHI* *chalcone-flavonone isomerase*, *CHS1* *chalcone synthase 1*, *CHS2* *chalcone synthase 2*, *DFR* *dihydroflavonol-4-reductase*, *F3H* *flavanone 3-hydroxylase*, *F3*′*H* *flavonoid 3*′*-hydroxylase*, *3-GT* *flavonol-3-glucosyltransferase*. **d** Anthocyanin accumulation in *Slhy5* mutants during fruit ripening. The error bars represent the standard deviations from three independent experiments. The asterisks indicate significant differences (*P < 0.05)

HY5 has been demonstrated to regulate anthocyanin biosynthesis in tomato and *Arabidopsis*^22,23^. Anthocyanin biosynthesis via the phenylpropanoid pathway is a complicated secondary metabolic process^24^, and chalcone synthase (CHS), chalcone isomerase (CHI), and flavanone 3-hydroxylase (F3H) contribute to the early steps of anthocyanin biosynthesis. Flavonoid 3′-hydroxylase (F3′H), a P450 enzyme, catalyzes the hydroxylation of dihydrokaempferol to form dihydroquercetin. Dihydrokaempferol and dihydroquercetin are then reduced to the corresponding leucoanthocyanidins by dihydroflavonol-4-reductase (DFR). Anthocyanins are converted from leucoanthocyanins by anthocyanidin synthase (ANS) and UDP-glucose:flavonoid-O-glycosyltransferase (3-GT). Our RNA-seq data showed that crucial genes involved in anthocyanin biosynthesis (*CHS1*, *CHS2*, *CHI*, *F3H*, *F3*′*H*, *DFR*, *ANS*, and *3-GT*) were markedly reduced in the *Slhy5* mutants (Fig. 4a, b), and a ChIP-qPCR analysis indicated that all of these genes were directly regulated by SlHY5 (Fig. 4c).

We then measured anthocyanin content in the wild-type and *Slhy5* mutant fruits at various ripening stages. During the early ripening stages (36 DPA and 39 DPA), no significant difference in the anthocyanin content was found between the wild-type and *Slhy5* mutants. However, at the later ripening stage (45 DPA), the accumulation of anthocyanin in the *Slhy5* mutants was distinctly lower than that in the wild type (Fig. 4d). These results confirmed that SlHY5 regulates anthocyanin biosynthesis directly.

### *SlHY5* mutation influences ethylene production and response

**Fig. 5:**
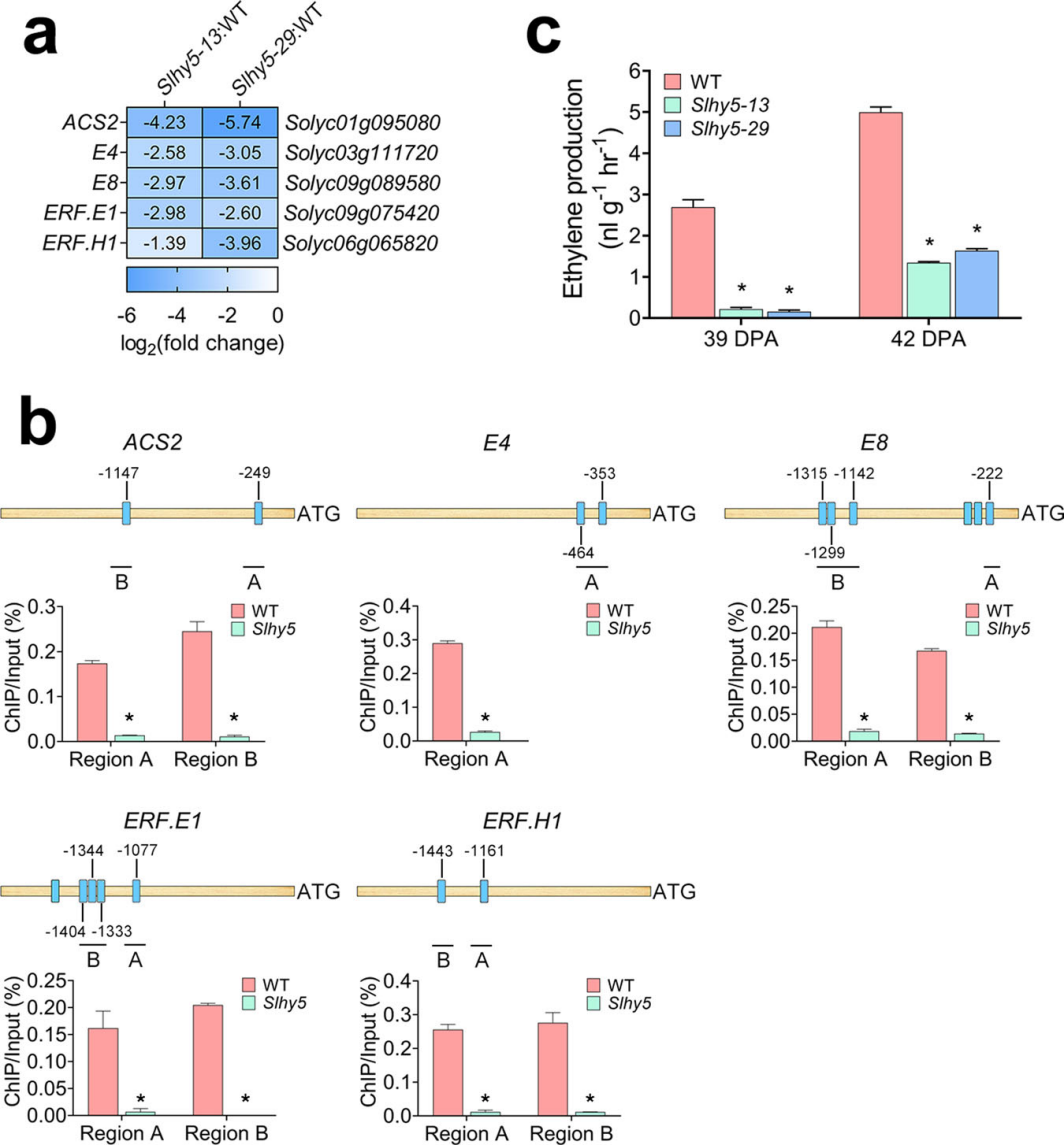
Ethylene production and response are regulated by SlHY5. **a** Heatmap showing the differentially expressed genes involved in ethylene production and response identified from the comparison of the *Slhy5* mutant (*Slhy5-13* and *Slhy5-29* lines) versus wild-type (WT) fruits at 39 days post anthesis (DPA) based on RNA-seq analysis. **b** ChIP-qPCR assays showing that SlHY5 binds to the promoters of genes involved in ethylene production and response. The promoter structures of the SlHY5 target genes are shown. The blue boxes represent ACGT-containing elements, and the numbers indicate the positions of these motifs relative to the translational start site. The black lines with uppercase letters represent the regions used for ChIP-qPCR. The values are the percentages of DNA fragments that coimmunoprecipitated with anti-SlHY5 antibodies relative to the input DNAs. *ACS2* *1-aminocyclopropane-1-carboxylic acid synthase 2*, *E4* *ethylene response gene 4*, *E8* *ethylene response gene 8*, *ERF.E1* *ethylene response factor E1*, *ERF.H1* *ethylene response factor H1*. **c** Ethylene release in *Slhy5* mutants at 39 and 42 DPA. The error bars represent the standard deviations from three independent experiments. The asterisks indicate significant differences (*P < 0.05)

The RNA-seq analysis showed that a number of genes involved in ethylene biosynthesis and response were downregulated in the *Slhy5* mutants (Fig. 5a and Supplementary Fig. 4). Five of these genes (*ACS2*, *E4*, *E8*, *ERF.E1*, and *ERF.H1*) were selected for the ChIP-qPCR assay due to their critical roles in ethylene signaling. *ACS* encodes 1-aminocyclopropane-1-carboxylic acid (ACC) synthase, which is the rate-limiting enzyme in ethylene biosynthesis. Fourteen ACS isoforms have been identified in tomato^25^. ACS2 represents the most abundant ACS isoform in ripening fruit, and the inhibition of *ACS2* by antisense RNA blocks fruit ripening^26^. A ChIP-qPCR assay showed that SlHY5 directly bound to the promoter of *ACS2* (Fig. 5b).

*E4* encodes a putative methionine sulfoxide reductase, and *E8* encodes a 1-aminocyclopropane-1-carboxylate oxidase homolog without catalytic activity to convert ACC to ethylene^27,28^. Although the molecular functions of *E4* and *E8* remain unclear, they are considered typical ripening-related genes^29,30^. As shown in Fig. 5b, both *E4* and *E8* were directly targeted by SlHY5.

*SlERF.E1*, which was formerly known as *LeERF2* (ref. ^31^), encodes an ethylene response factor that displays a ripening-related expression pattern^32^, whereas *SlERF.H1*, previously named *LeERF1*, affects tomato fruit ripening and softening^33^. ChIP-qPCR assays indicated that SlHY5 directly bound to the promoters of *SlERF.E1* and *SlERF.H1* (Fig. 5b).

We subsequently analyzed ethylene synthesis and found that the *Slhy5* mutants produced appreciably decreased levels of ethylene than the wild-type fruits at 39 DPA and 42 DPA, when the ethylene burst arose (Fig. 5c). These data demonstrated that SlHY5 directly regulates ethylene production and response.

### Translation is partially impaired in the *Slhy5* mutants

**Fig. 6:**
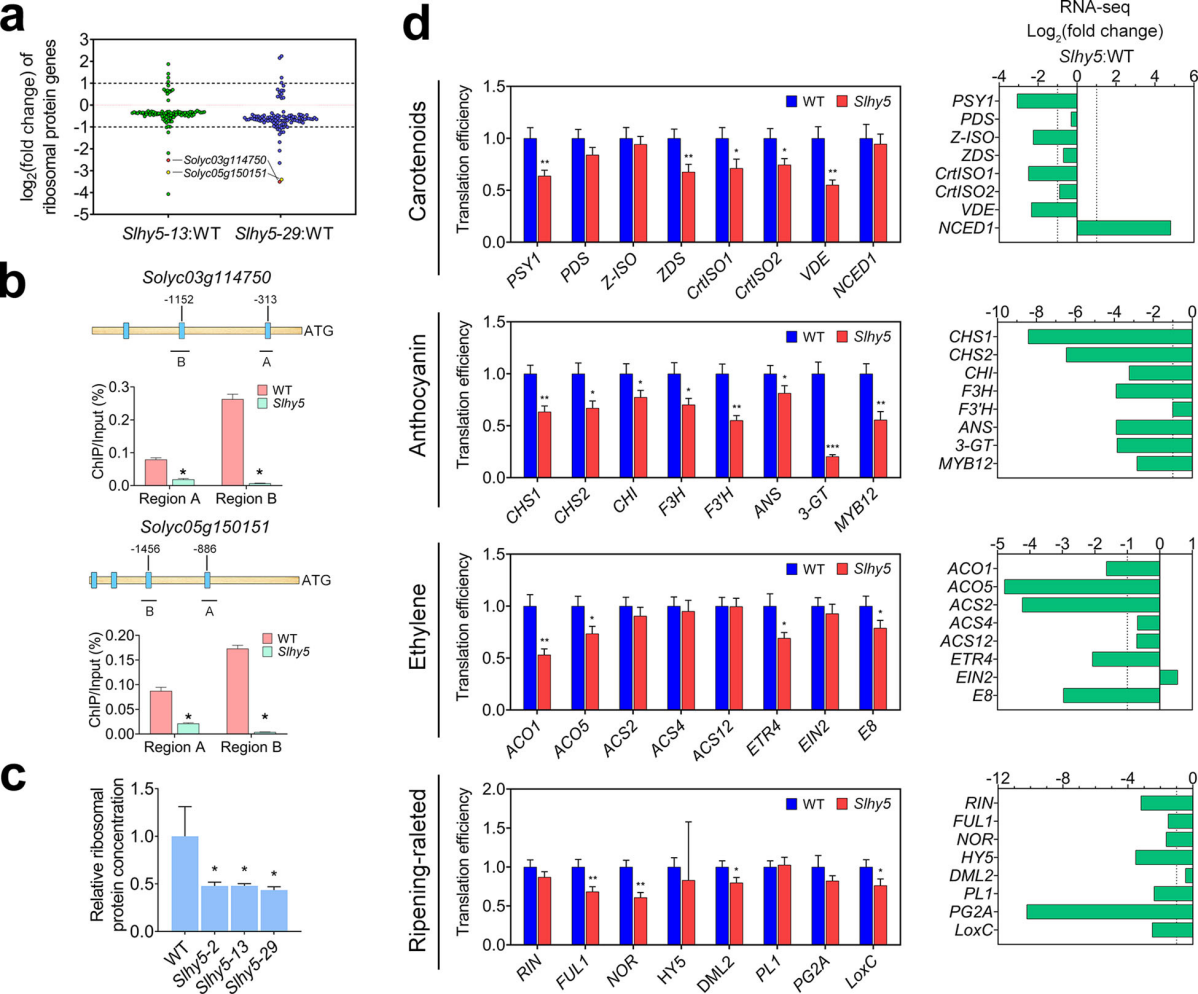
Protein translation is affected in the *Slhy5* mutant. **a** Differential expression of ribosomal protein genes in the *Slhy5* mutant (*Slhy5-13* and *Slhy5-29*) versus wild-type (WT) fruits at 39 days post anthesis (DPA) based on RNA-seq data. **b** ChIP-qPCR assays showing that SlHY5 binds to the promoters of two ribosomal protein genes. The promoter structures of the SlHY5 target genes are shown. The blue boxes represent ACGT-containing elements, and the numbers indicate the positions of these motifs relative to the translational start site. The black lines with uppercase letters represent the regions used for ChIP-qPCR. The values are the percentages of DNA fragments that coimmunoprecipitated with anti-SlHY5 antibodies relative to the input DNAs. **c** Relative ribosomal protein concentration in *Slhy5* mutant compared with WT fruits at 39 DPA. The error bars represent the standard deviations from three independent experiments. The asterisks indicate significant differences (*P < 0.05). **d** Translation efficiency of specific genes was altered in the *Slhy5* mutants. *PSY1* *phytoene synthase 1*, *PDS* *phytoene desaturase*, *Z-ISO* *15-cis-zeta-carotene isomerase*, *ZDS* *zeta-carotene desaturase*, *CrtISO1* *carotene isomerase 1*, *CrtISO2* *carotene isomerase 2*, *VDE* *violaxanthin de-epoxidase*, *NCED1* *9-cis-epoxycarotenoid dioxygenase 1*, *ANS* *anthocyanidin synthase*, *CHI* *chalcone-flavonone isomerase*, *CHS1* *chalcone synthase 1*, *CHS2* *chalcone synthase 2*, *F3H* *flavanone 3-hydroxylase*, *F3*′*H* *flavonoid 3*′*-hydroxylase*, *3-GT* *flavonol-3-glucosyltransferase*, *MYB12 MYB transcription factor 12*, *ACO1* *1-aminocyclopropane-1-carboxylate oxidase 1*, *ACO5* *1-aminocyclopropane-1-carboxylate oxidase 5*, *ACS2* *1-aminocyclopropane-1-carboxylic acid synthase 2*, *ACS4* *1-aminocyclopropane-1-carboxylic acid synthase 4*, *ACS12* *1-aminocyclopropane-1-carboxylic acid synthase 12*, *ETR4* *ethylene receptor 4*, *EIN2* *ethylene insensitive 2*, *E8* *ethylene response gene 8*, *RIN* *ripening inhibitor*, *FUL1* *FRUITFULL 1*, *NOR* *non-ripening*, *HY5* *ELONGATED HYPOCOTYL5*, *DML2* *DEMETER-like DNA demethylase 2*, *PL1* *pectate lyase 1*, *PG2A* *polygalacturonase 2A*, *LoxC* *lipoxygenase C*. The error bars represent the standard deviations from three independent experiments. The asterisks indicate significant differences (*P < 0.05, **P < 0.01, ***P < 0.001; Student’s *t* test). The corresponding transcriptional levels of these genes are listed in the right panel

According to International Tomato Annotation Group version 4.0 (ITAG4.0), 544 annotated genes encode ribosomal proteins. Interestingly, in our RNA-seq analysis, we identified 107 genes encoding ribosomal proteins (129 identified in *Slhy5-13* and 219 identified in *Slhy5-29*) whose expression was significantly altered (*P-*adj < 0.05) in both *Slhy5* mutants (Fig. 6a and Supplementary Table 3). Most of these genes (86.0%) showed decreased expression in the mutants. We selected six genes with a fold change > 2 for the ChIP-qPCR assay, and the results indicated that two of these genes (*Solyc03g114750* and *Solyc05g150151*) are direct targets of SlHY5 (Fig. 6b).

A decrease in the expression of genes encoding ribosomal proteins might influence the pool of ribosomal proteins. We subsequently compared the abundances of ribosomal proteins, which were calculated from purified polysomes in the wild-type and *Slhy5* mutant fruits. The concentration of total soluble proteins prior to polysome enrichment was used as a control. More ribosomal proteins were enriched in the wild-type than in the *Slhy5* mutant fruits (Fig. 6c), which indicated that loss of function of *SlHY5* affects the accumulation of ribosomal proteins in ripening fruit.

Since ribosomal proteins are core components of the ribosome, which is a protein translation machinery, we speculate that SlHY5 might affect protein translation. We next determined the translation efficiency by calculating the ratio of the expression level of specific genes in polysomal to total RNA. Genes involved in various metabolic processes, including carotenoid metabolism, anthocyanin accumulation, ethylene biosynthesis and response, and some well-known ripening-related genes were evaluated. As shown in Fig. 6d, the translation efficiency of all the detected genes associated with anthocyanin accumulation was decreased in the *Slhy5* mutants. Approximately half of the tested genes involved in carotenoid metabolism and ethylene biosynthesis and response exhibited a reduced translation efficiency in the mutants. Four of the ripening-related genes, i.e., *FUL1*, *NOR*, *DML2*, and *LoxC*, displayed a reduced translation efficiency in the *Slhy5* mutants. It is worth noting that some genes (*CHS1*, *CHS2*, *CHI*, *F3H*, *F3’H*, *ANS*, *3-GT*, *MYB12*, *ACO1*, *ACO5*, *ETR4*, *E8*, *PSY1*, *CrtISO1*, *VDE*, *FUL1*, *NOR*, and *LoxC*) were regulated by SlHY5 at both the transcriptional and translational levels, whereas the others were regulated only at the transcriptional level (*ACS2*, *Z-ISO*, *NCED1*, *RIN*, *PL1*, and *PG2A*) or translational level (*ZDS*, *CrtISO2*, and *DML2*). These results suggest that SlHY5 has the ability to regulate gene expression not only at the transcriptional level, but also at the translational level during fruit ripening.

## Discussion

### SlHY5 regulates tomato fruit ripening at the transcriptional level

HY5 is well known for its function in the light signaling pathway. HY5 was first identified in *Arabidopsis* as a transcriptional regulator with a bZIP motif^9^. HY5 can bind up to eight types of *cis*-elements present in the promoters of the target genes, particularly G-box (CACGTG) and ACE-box (ACGT). Large-scale identifications of direct targets of HY5 have revealed that HY5 affects the expression of one-third of genes in the *Arabidopsis* genome and directly controls ~3000 of these genes^20,21^. In contrast to the understanding of HY5 in the model plant *Arabidopsis*, the functions and regulatory mechanisms of HY5 in the physiological processes of horticultural crops remain largely uncharacterized. In this study, we found that ~3000 genes were differentially expressed in the fruit of the *Slhy5* mutants (Fig. 2), which suggested that SlHY5 plays a global regulatory role in the process of tomato fruit ripening. ChIP-qPCR assays showed that genes involved in carotenoid metabolism and anthocyanin biosynthesis were directly regulated by SlHY5 (Figs. 3 and 4). All of these genes were downregulated in the *Slhy5* mutants, consistent with the results from the phenotypic analysis, which showed that *Slhy5* mutants contain lower levels of carotenoids and anthocyanins than wild-type fruits.

Intriguingly, genes involved in ethylene production and response were identified as direct targets of SlHY5 in tomato (Fig. 5). Consistent with the downregulation of these genes, ethylene production was substantially reduced in *Slhy5* mutants at 39 and 42 DPA (Fig. 5), which demonstrated that SlHY5 positively affects ethylene biosynthesis. Ethylene is necessary for climacteric fruit ripening. As a classic climacteric fruit, tomato shows a burst of ethylene at the onset of fruit ripening^34^. These data indicated that SlHY5 is necessary for normal fruit ripening in tomato. SlHY5 might influence fruit ripening directly by binding to the promoters of ripening-related genes, as observed with carotenoid biosynthetic genes, or indirectly by affecting ethylene signaling. Notably, *Arabidopsis* seedlings with *hy5* mutation showed 1.5-fold higher ethylene emissions than wild-type seedlings, which implies that HY5 exerts a negative effect on ethylene production^35^. A similar antagonistic relationship between HY5 and ethylene generation has also been observed on hypocotyl growth in *Arabidopsis*, and exogenous ethylene can promote HY5 degradation^36^. The discrepancy could be explained by the difference between seedlings/hypocotyls and fruit, in which ethylene plays different roles.

As a programmed developmental process, fruit ripening involves epigenetic regulations and the coordination of plant hormones and transcription factors^29,34,37–40^. A number of transcription factors, including RIN, NOR, CNR, TAGL1, and FUL1/FUL2, have been characterized to be involved in fruit ripening^29,37,38,41^. In the present study, we found that the expression of *RIN*, *CNR*, and *FUL1* was reduced in the *Slhy5* mutants. Further ChIP-qPCR analysis revealed that SlHY5 bound to the promoters of *RIN*, *CNR*, and *FUL1*, which indicates that SlHY5 directly regulates these transcription factors (Supplementary Fig. 5). The interplay between SlHY5 and these ripening regulators deserves further investigation.

Notably, HY5 regulates various physiological processes of plants^9,14,15^, such as seedling growth, leaf development, and flowering, which may influence fruit physiology. SlHY5-mediated fruit ripening might be partly influenced by these developmental processes.

### SlHY5 regulates the translation efficiency of numerous ripening-related genes

It has been elucidated that light modulates gene expression at multiple levels. In addition to the transcriptional regulation mediated by transcription factors, posttranscriptional regulation, such as alternative splicing and selected degradation of mRNA via small regulatory RNAs, has been demonstrated to attenuate the expression of light-responsive genes^21,42^. Light also regulates gene expression at the posttranslational level through the ubiquitin-dependent protein degradation of key components in the light signaling pathway or by affecting protein phosphorylation/dephosphorylation^43^. Furthermore, light regulates the translation of expressed genes in plants^44^. A global survey of transcripts under translational control during photomorphogenesis in *Arabidopsis* was recently performed^45^. Information about translational regulation offers knowledge on how an organism copes with intrinsic development cues and responds to external environmental stimuli^45^. Although a large number of genes are regulated at the translational level in plants^45^, the linkers connecting the light signals and the downstream translational regulation of gene expression have not been defined.

In the present study, we found that a large portion of genes encoding ribosomal proteins were downregulated in *Slhy5* mutants (Fig. 6a), and several of these ribosomal protein genes were identified as direct targets of SlHY5 (Fig. 6b). Ribosomal proteins represent the core components of the translation machinery. A lack of ribosomal proteins causes an insufficient ribosome pool and impairs the translation of transcripts. We further demonstrated that the translation efficiency of multiple ripening-related genes was decreased in the *Slhy5* mutants (Fig. 6d), which indicated that SlHY5 affects the translation of these genes. Interestingly, no significant changes in mRNA levels have been observed for some of these ripening-related genes (e.g., *ZDS* and *DML2*) in the *Slhy5* mutants, which indicates that these genes are specifically regulated at translational level. Our data suggest that HY5 might serve as the linker in the light-regulated translational control of gene expression.

**Fig. 7:**
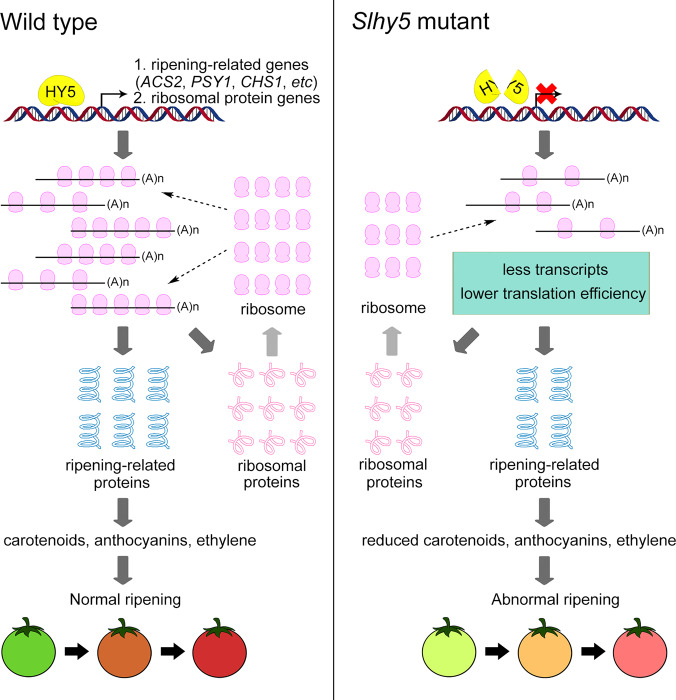
Model of the regulatory role of SlHY5 in tomato fruit ripening. SlHY5 regulates fruit ripening at both the transcriptional and translational levels. SlHY5 can bind directly to the promoters of genes involved in pigment accumulation, ethylene production and response, and translation to modulate their expression at the transcriptional level. SlHY5 also regulates the translation efficiency of ripening-related genes by targeting ribosomal protein genes. Loss of function of *SlHY5* influences the transcription and translation of ripening-related genes, which leads to delayed fruit ripening

In summary, we demonstrated that SlHY5 participates in tomato fruit ripening through the transcriptional regulation of ripening-related genes, such as those involved in carotenoid biosynthesis and ethylene signaling. We also proved that SlHY5 affects the translation efficiency of a set of ripening-related genes by targeting ribosomal protein genes (Fig. 7). Our findings provide insight into the molecular regulatory network of fruit ripening and the multifaceted regulation of gene expression by transcription factors. Considering the conservation and vital role of HY5 in the light signaling pathway, we propose that the regulatory mechanisms described in this manuscript are also involved in the regulation of other light-controlled developmental processes.

## Materials and methods

### Plant materials

Wild-type tomato seeds (*S. lycopersicum* cv. Ailsa Craig) were kindly provided by Dr. James J. Giovonnoni (Boyce Thompson Institute for Plant Research, Cornell University, Ithaca, NY, USA). The tomato plants were grown in a greenhouse under standard culture conditions. Flowers were tagged on the day of anthesis to evaluate the ripening stages of the fruits. Wild-type fruits were harvested at the mature green (MG), breaker (Br), orange (Or), and red ripe (RR) stages, which occurred on average at 36, 39, 42, and 45 DPA. Fruits from the *Slhy5* mutants were harvested at equivalent ripening stages, as determined by the number of DPA. Upon harvesting, the pericarps were collected, frozen immediately in liquid nitrogen, and stored at −80 °C until use.

### Construction of *Slhy5* mutants using the CRISPR/Cas9 system

A highly efficient CRISPR/Cas9 system improved for plant genome editing^46^ was used in this study. CRISPR-P (http://cbi.hzau.edu.cn/crispr/), an online tool, was used to generate a pool of target sequences, and we selected two target sites for *SlHY5* based on previously reported principles^46^. Once the target sequences were selected, a PCR-based method was applied to rapidly generate multiple sgRNA expression cassettes that could be easily cloned into the pYLCRISPR/Cas9Pubi-H binary vector by Golden Gate ligation. The final construct pYLCRISPR/Cas9Pubi-H-*SlHY5* was sequenced and then transformed into wild-type tomato through *A. tumefaciens*-mediated transformation.

After regeneration, tomato seedlings were transplanted into soil and cultured in a greenhouse. Genomic DNA of regeneration seedlings was extracted from fresh leaves using the FastPure Plant DNA Isolation Mini Kit (Vazyme, DC104). The presence of the transgene was verified by PCR to confirm the insertion of T-DNA. Subsequently, to validate the mutation of the transgenic plants, second-round PCR was performed using primers flanking the target sites. The PCR products were sequenced directly, and the superimposed sequencing chromatograms were decoded manually or using the automated web tool DSDecodeM (http://skl.scau.edu.cn/dsdecode/). Subsequently, the mutated and original sequences were aligned to show the mutagenesis.

### RNA extraction and quantitative real-time PCR

Pericarps of fruits (at least eight fruits) from six plants were used to extract total RNA, following a previously described method^47^. One microgram of total RNA was used for first-strand cDNA synthesis with a HiScript III RT SuperMix for qPCR kit (Vazyme, R323-01). Quantitative real-time PCR was performed with ChamQ Universal SYBR qPCR Master Mix (Vazyme, Q711), using the StepOnePlus Real-Time PCR System (Applied Biosystems). Gene expression levels were calculated using the 2^−ΔΔCt^ analysis method, and the samples were normalized using *ACTIN* (*Solyc11g005330*). Three distinct biological replicates of each experiment were included.

### RNA sequencing

For RNA-seq, pericarps of fruits (at least eight fruits) at 39 DPA from six wild-type and *Slhy5* mutant (*Slhy5-13* and *Slhy5-29*) plants were collected for total RNA isolation. RNA-seq libraries were constructed using the NEBNext^®^ Ultra^TM^ RNA Library Prep Kit for Illumina (NEB) and sequenced using a HiSeq PE150 instrument (Illumina). In total, ~500 million raw reads were obtained, and all the data were uploaded to the BIG Data Center Genome Sequence Archive (https://bigd.big.ac.cn/gsub/) under accession number CRA003226. The raw reads were filtered using FastQC to generate clean data, which were aligned to the tomato reference genome SL4.0 using HISAT2 (version 2.0.5). FeatureCounts (version 1.5.0-p3) was used to count the numbers of reads mapped to each gene, and RPKM values were calculated based on the length of each gene and the read count mapped to the gene. Differential expression analysis between the wild-type and *Slhy5* mutant plants was performed, using the DESeq2 R package (version 1.16.1). The resulting *P* values were adjusted using Benjamini and Hochberg’s approach for controlling the false discovery rate (the adjusted values are denoted *P*-adj hereafter). The fold changes were calculated using the formula FPKM_*Slhy5*_/FPKM_WT_. Transcripts satisfying the criteria |log_2_(fold change)| > 1 and *P*-adj < 0.05 were defined as differentially expressed.

### Ethylene production assay

To measure ethylene release, at least six fruits from six wild-type and *Slhy5* mutant plants (*Slhy5-13* and *Slhy5-29*) were harvested at 39 and 42 DPA. After harvest, the fruits were placed at room temperature for 3 h to dissipate the field heat and wound ethylene. The fruits were then transferred to jars, and the jars were then sealed and incubated for another 2 h. The gas samples were analyzed using a gas chromatograph (Agilent 7820 A). The production of ethylene was calculated through a comparison with the standard sample and normalization of the fruit weight.

### Determination of pigments

Total anthocyanins were extracted following a previously described method^48^ with some modifications. One gram of pericarp sample was ground into fine powder in liquid nitrogen and incubated in 3 ml of 1% HCl in methanol (v/v) overnight at room temperature. An additional 3 ml of chloroform was then added and mixed thoroughly to remove fat-soluble pigments. After centrifugation at 12,000 × *g* for 2 min, the supernatants were collected and filtered through a 0.45-μm filter. The content of anthocyanins was determined spectrophotometrically at 535 nm and expressed as *A*_535_ g^−1^ fresh weight. Average values were calculated from three independent replicates.

Carotenoids were extracted as described previously^49^. The carotenoid content was measured using a Waters ACQUITY UPC2 System (Milford, MA, USA) equipped with a C18 column, according to the manufacturer’s recommendations. The individual carotenoids (phytoene, lycopene, and β-carotene) were identified according to the retention time and quantified using standard curves.

### Recombinant SlHY5 protein expression and specific antibody preparation

The full-length *SlHY5* open reading frame was amplified from tomato cDNA using the primers HY5-F (CTTTAAGAAGGAGATATACATATGCACCATCATCATCATCATCAAGAGCAAGCGACGAGTTC) and HY5-R (GTGGTGGTGGTGGTGGTGCTCGAGCTACTTCCTCCCTTCCTGTG). The PCR product was inserted into the pET30a vector digested with Nde I and Xho I using a ClonExpress Ultra One Step Cloning Kit (Vazyme, C115) to generate pET30a-6×His-SlHY5. The recombinant plasmid was transformed into *Escherichia coli* BL21 (DE3) competent cells, and the expression of the recombinant protein was then induced with 1 mM isopropyl-1-thio-β-d-galactopyranoside. The recombinant protein was purified using Ni-NTA His Bind Resin (Merck, 70666) following the manufacturer’s instructions, and rabbits were immunized with the purified recombinant protein at Beijing Protein Innovation Company (http://www.proteomics.org.cn/). Polyclonal antibody was affinity purified from antisera using AminoLink Plus Coupling Resin according to the manual (Thermo Scientific, 20501).

### Nuclei enrichment

Nuclei were isolated from tomato fruit at 39 DPA according to a previously described method^47^. Briefly, the pericarp was ground into fine powder in liquid nitrogen and suspended in buffer 1 (0.4 M sucrose, 10 mM Tris-HCl, pH 8.0, 5 mM β-mercaptoethanol, and 1 mM PMSF). The homogenates were filtered through two layers of miracloth and centrifuged at 3000 × *g* for 20 min. The pellets were suspended softly in buffer 2 (0.25 M sucrose, 10 mM Tris-HCl, pH 8.0, 10 mM MgCl_2_, 1% Triton X-100, 5 mM β-mercaptoethanol, and 1 mM PMSF) and set aside on ice for 10 min. After centrifugation at 12,000 × *g* for 10 min, the pellets were resuspended in 500 μl of buffer 3 (1.7 M sucrose, 10 mM Tris-HCl, pH 8.0, 2 mM MgCl_2_, 0.15% Triton X-100, 5 mM β-mercaptoethanol, and 1 mM PMSF) and overlaid on top of another 500 μl of buffer 3. The nuclei were enriched by centrifugation at 16,000 × *g* for 45 min and stored at −80 °C until use.

### Nuclear protein extraction and western blotting

The purified nuclei were thoroughly resuspended in extraction buffer (0.7 M Sucrose, 0.5 M Tris-HCl, pH 8.0, 0.5 M EDTA, 0.1 M KCl, 5 mM β-mercaptoethanol, and 1 mM PMSF) and broken by sonication on ice. The homogenate was mixed with an equal volume of Tris-HCl saturated-phenol (pH 7.4) and vortexed for 10 min at 4 °C. After centrifugation at 20,000 × *g* for 15 min, the upper-layer phenol phase was re-extracted with extraction buffer. The proteins in the phenol phase were precipitated by adding five volumes of methanol containing 0.1 M ammonium acetate and stored overnight at −20 °C. The protein pellets were collected by centrifugation at 20,000 × *g* for 15 min, and washed several times with ice-cold methanol and acetone. The nuclear proteins were air-dried and dissolved in 0.5 M Tris-HCl (pH 8.0) containing 1% SDS.

For immunoblot analysis, 15 μg of nuclear proteins was separated by 12% SDS–PAGE and then transferred to an Immobilon-P PVDF membrane (Millipore, IPVH00005) by semidry transfer (Amersham Biosciences, TE77 PWR). The membranes were blocked with 5% nonfat milk in TBS-T buffer for 2 h at room temperature, and incubated with an anti-SlHY5 (1:1000) or anti-histone H3 (1:10000) antibody for 1 h. The membranes were then washed four times with TBS-T buffer and incubated with the secondary antibody (1:5000) for 1 h. The membranes were washed four times with TBS-T buffer and visualized using a chemiluminescence detection kit (SuperSignal, Pierce Biotechnology).

### Chromatin immunoprecipitation

ChIP assays were performed as described previously with some modifications^47^. Pericarp tissues of fruits (at least eight fruits) at 39 DPA from six wild-type and *Slhy5-13* mutant plants were sliced and submerged in 1% formaldehyde under vacuum for 20 min for the cross-linking of genomic DNA and protein. The fixed materials were subjected to nuclear isolation as described above. The enriched nuclei were sonicated to shear gDNA to an average size of 300–800 bp. After centrifugation at 12,000 × *g* for 2 min, the supernatant, which contained the sheared chromatin, was collected. A portion of the supernatant was reverse cross-linked and used as the input DNA control. The affinity-purified anti-SlHY5 antibody was incubated with preblocked Magna ChIP^TM^ Protein A + G Magnetic Beads (Millipore, 16-663) overnight at 4 °C, and the remaining chromatin sample was then added. After incubation for 4 h, the beads were washed successively with low-salt wash buffer, high-salt wash buffer, lithium chloride wash buffer, and TE buffer. The DNA–protein complexes were eluted from the beads by occasional rotation at 65 °C for 1 h. The cross-linking between immunoprecipitated DNA and SlHY5 protein was interrupted by adding NaCl to a final concentration of 0.2 M, and the eluate was incubated overnight at 65 °C. The immunoprecipitated DNA was purified using a TIANquick Mini Purification Kit (TIANGEN, DP203). The HY5-binding sites (ACGT-containing elements) in the promoters of the candidate genes were analyzed with PlantPAN 3.0 (http://plantpan.itps.ncku.edu.tw/TFsearch.php). The primers used for quantitative PCR were designed using Primer Premier 5 software and are listed in Supplementary Table 4.

### Translation efficiency and ribosomal protein determination

The translation efficiency was assayed following a previously described method with some modifications^50^. All procedures were operated on ice or at 4 °C. Pericarps of fruits (at least eight fruits) at 39 DPA from six wild-type and *Slhy5-13* mutant plants were ground into fine powder in liquid nitrogen. One gram of sample was used for total RNA extraction, and the remaining sample was suspended in 15 ml of polysome extraction buffer (200 mM Tris-HCl, pH 9.0, 200 mM KCl, 35 mM MgCl_2_, 25 mM EGTA, 1% Triton X-100, 1% IGEPAL CA-630, 5 mM DTT, 1 mM PMSF, 100 μg/ml cycloheximide, and 50 μg/ml chloramphenicol) and maintained on ice for 10 min. The extract was filtered through two-layer miracloth and then centrifuged at 16,000 × *g* for 15 min. Exactly 10 ml of the supernatant was loaded on a sucrose cushion (1.75 M sucrose, 400 mM Tris-HCl, pH 9.0, 200 mM KCl, 35 mM MgCl_2_, 5 mM EGTA, 5 mM DTT, 50 μg/ml cycloheximide, and 50 μg/ml chloramphenicol), and the remaining supernatant was reserved for total soluble protein determination. Polysomes were collected after ultracentrifugation at 190,000 × *g* for 4 h and resuspended in 500 μl of RNase-free ddH_2_O (50 μl was reserved for ribosomal protein determination). RNA in the polysomes were extracted using the hot phenol method. The relative expression of the target genes in total or polysomal RNA was calculated using the 2^−ΔΔCt^ analysis method, and the samples were normalized using *ACTIN*. The translation efficiency was calculated as the relative expression in polysomal/total RNA followed by normalization against that in the wild-type fruits. The primers used for quantitative PCR were designed using Primer Premier 5 software and are listed in Supplementary Table 5.

The total soluble protein (TSP) and ribosomal protein (RP) concentrations were determined using a Pierce^TM^ BCA Protein Assay Kit (Thermo, 23225). The relative RP content was calculated using the formula Concentration_(RP)_/Concentration_(TSP)_ followed by normalization against that of the wild-type plants.

## Supplementary information

Supplementary Information (Fig. S1-S5, Table S4-S5)

Supplementary Information (Table S1-S3)
