# Supplementary material for: The transcription factor SlHY5 regulates the ripening of tomato fruit at both the transcriptional and translational levels
Source: Hortic Res. 2021 Apr 1;8:83. doi: 10.1038/s41438-021-00523-0 (PMC8012583; doi:10.1038/s41438-021-00523-0)
Supplement: Supplementary file 1 — Supplementary Information (Fig. S1-S5, Table S4-S5) [file 41438_2021_523_MOESM1_ESM.docx]

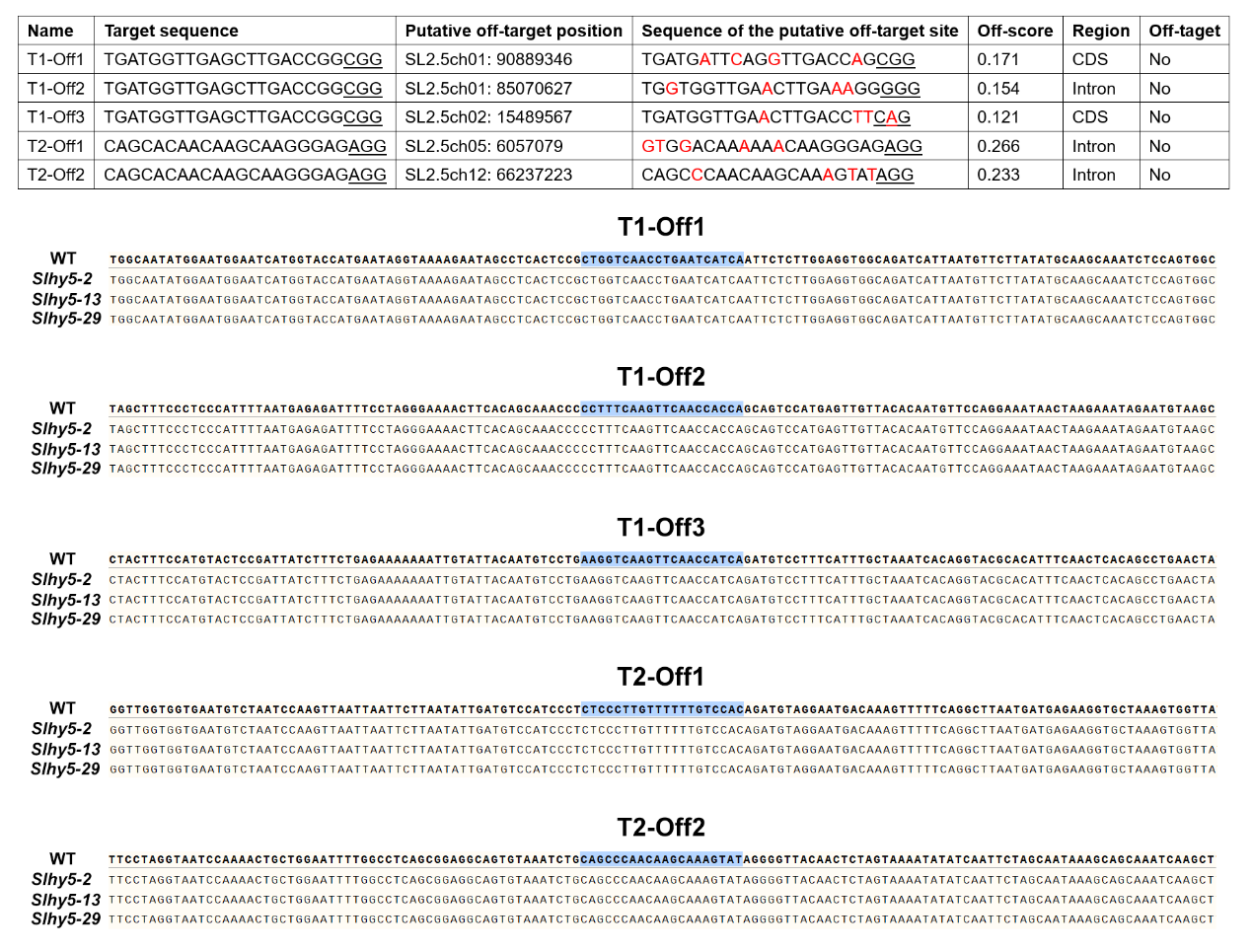


Supplementary Fig. 1 Off-target analysis in *Slhy5-2*, *Slhy5-13*, and *Slhy5-29* mutants. Potential off-target sites were predicted by CRISPR-P (http://crispr.hzau.edu.cn/CRISPR/). Underlined letters indicate the protospacer adjacent motif (PAM). Red letters refer to the mismatched nucleotides between off-target sites and target sequences. The potential off-target sites were sequenced using PCR products from genomic DNA flanking these sites. Sequence alignment around the potential off-target sites in wild-type (WT) with those of *Slhy5* mutants are shown. The sequences of potential off-target sites were highlighted with blue background.


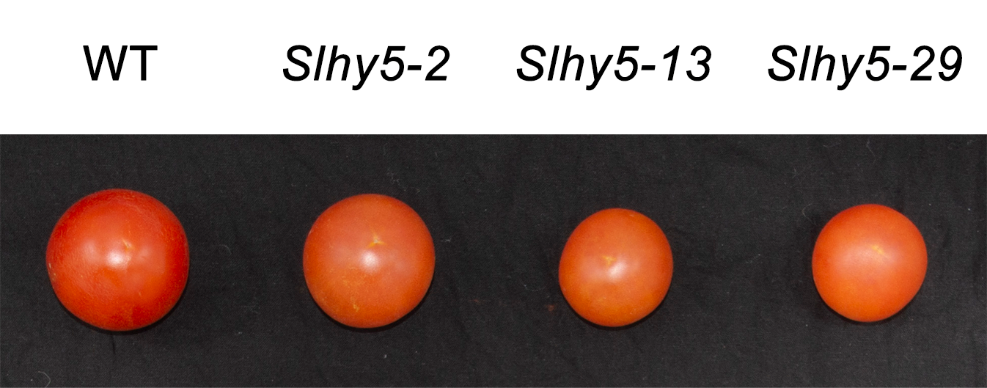


Supplementary Fig. 2 Phenotype of over-ripe fruit from tomato at 52 days post anthesis (DPA). Over-ripe fruit of *Slhy5* mutants shows light red color, while fruit of wild-type (WT) displays vivid red color.


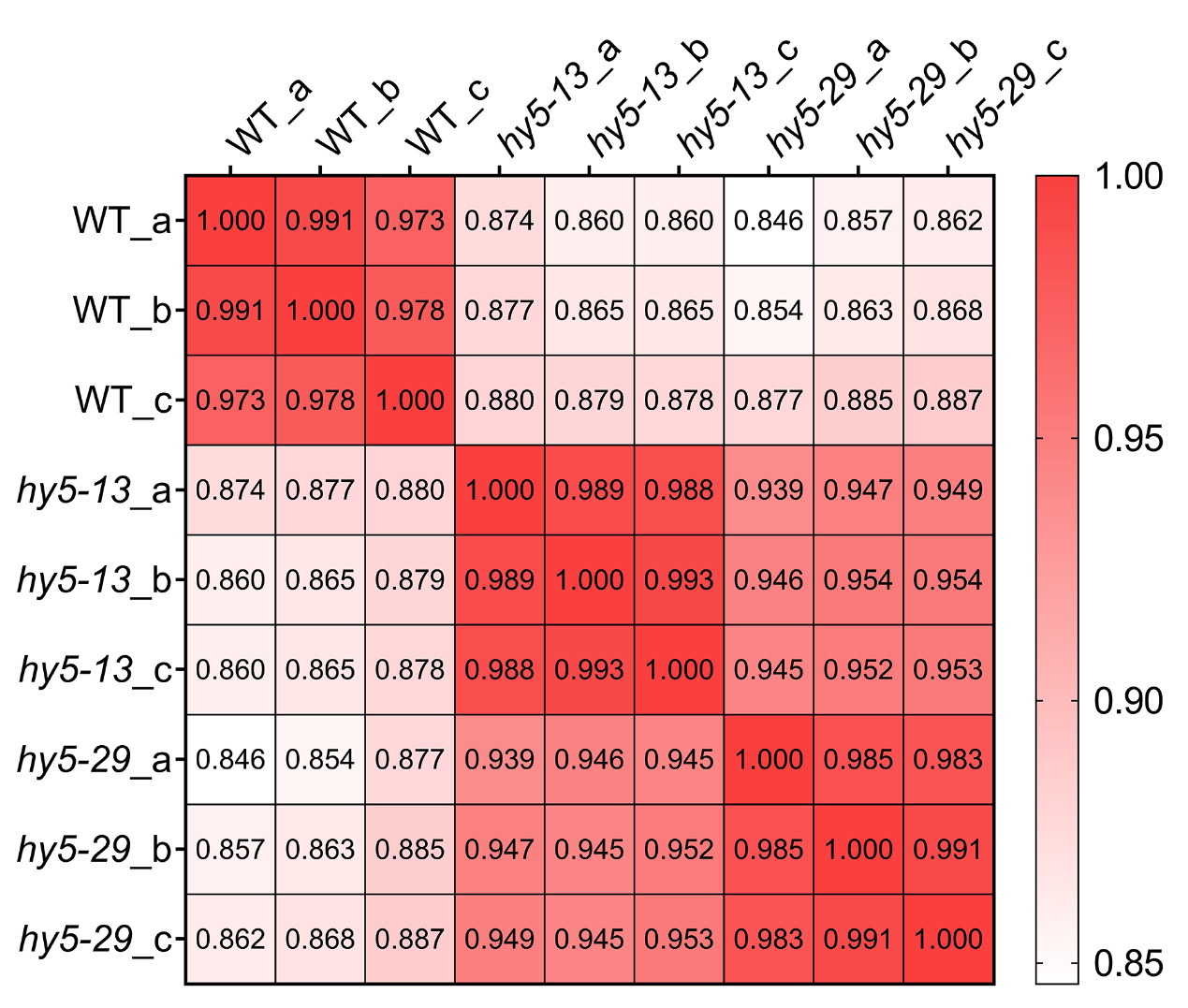


Supplementary Fig. 3 Pearson correlation between samples in RNA-seq. The a, b, c suffixes mean the three replicates. Numbers in the cells represent the square of Pearson correlation coefficient between two samples.


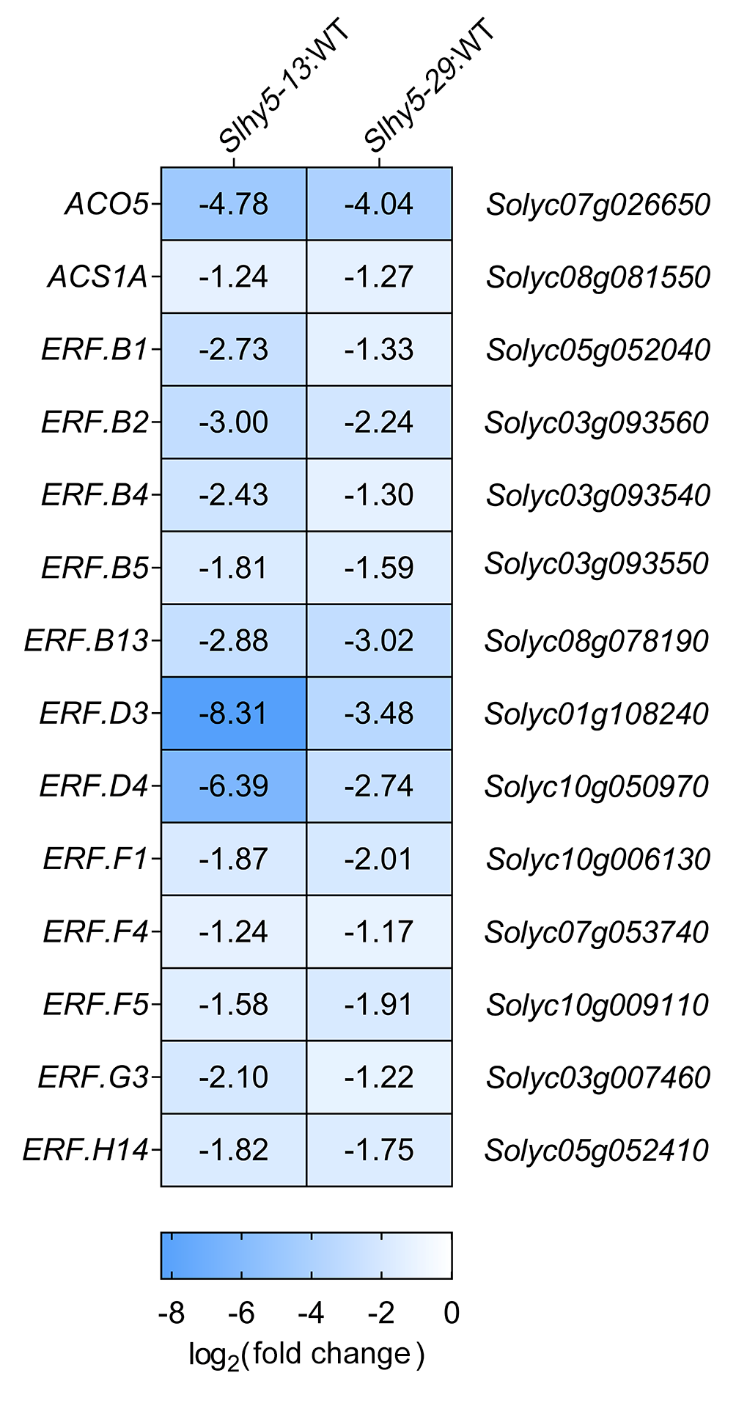


Supplementary Fig. 4. Heat map showing the expression of genes involved in ethylene production and response in *Slhy5* mutants (*Slhy5-13* and *Slhy5-29*) based on RNA-seq data. *ACO5*, *1-aminocyclopropane-1-carboxylate oxidase 5*; ACS1A, 1-aminocyclopropane-1-carboxylic acid synthase 1A; *ERF*, *Ethylene response factor*.


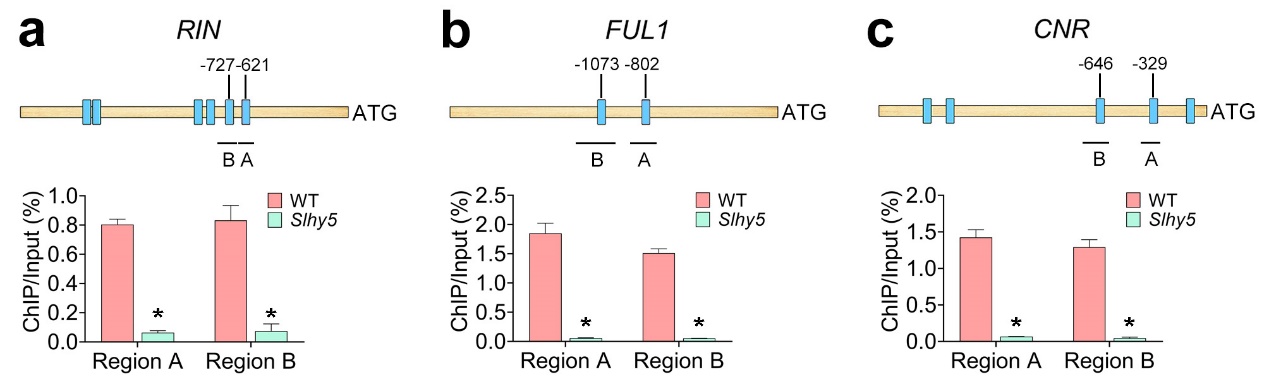


Supplementary Fig. 5 ChIP-qPCR assays showing that SlHY5 binds to the promoters of ripening-related transcription factors *RIN* (**a**), *FUL1* (**b**), *CNR* (**c**). The promoter structures of the SlHY5 target genes are shown. Blue boxes represent ACGT-containing elements and numbers indicate the position of these motifs relative to the translational start site. Black lines with upper-case letters represent the regions used for ChIP-qPCR. Values are the percentage of DNA fragments that co-immunoprecipitated with anti-SlHY5 antibodies relative to the input DNAs. *RIN*, *Ripening inhibitor*; *FUL1*, *FRUITFULL 1*; *CNR*, *Colorless non-ripening*. Error bars represent the standard deviation of three independent experiments. Asterisks indicate significant differences (**P* < 0.05).

**Supplementary Table 4. Primers used in ChIP-qPCR assay.**

| **Primer name** | **Primer sequence (5'→3')** |
| --- | --- |
| CHS1-FA | GATCGTTGATCTTGATAAAGTAGGT |
| CHS1-RA | GAAAGAGTGATAATTGTCTTGGTGT |
| CHS1-FB | GATTGAGAAGTTTAATCCGTATCGA |
| CHS1-RB | GCTACCTACTTTATCAAGATCAACG |
| CHS2-FA | CGAACAACTCCGCTCGTCGATTAC |
| CHS2-RA | TGGCTTGTGTTAAGTTTGCATGGAG |
| CHS2-FB | CGGTGGTTACGTCTTAAATTGGATA |
| CHS2-RB | CTATTTGCCACTAATGCTACCTACT |
| CHI-FA | TCTTATCCCACCGAAAAACGACGTA |
| CHI-RA | GCTACCACACAATCTACCTTCCAGC |
| CHI-FB | TATCATCCGAACAACTCCGCTC |
| CHI-RB | CATAACAAACTGGTAGGCGGTAGC |
| F3H-FA | CAAGTATGGTGGTACCTACCCTAC |
| F3H-RA | GCTATTACTACCCTTCGTGCCAACT |
| F3H-FB | AGGCAACGCTGGTTGGTAATAGTC |
| F3H-RB | ACGGAGGGTGAATGAAATGGAATG |
| F3'H-FA | TACTAAAATTCACTTGTGCCCGATG |
| F3'H-RA | GAATGTGTCATGATCAAATTAACGG |
| F3'H-FB | ACCAAGCAATGCAACTGATTACATG |
| F3'H-RB | CGAATAGAGATCCTTTAGTTATTAGCTG |
| DFR-FA | GCTTCATGGAGCGGACTTGTCA |
| DFR-RA | GAGGTCCATCTTGATCGCACGT |
| DFR-FB | GCAAGTAAAGTGGCAGAAGCAC |
| DFR-RB | AAGAGGGCTTTGATACCCAGAC |
| 3GT-FA | ATGACACGTTTATGCGTTATAGGTG |
| 3GT-RA | CCTCGTGGACGAATTATCAGCC |
| ANS-FA | CCCAAAGAACTTGTAACAGTTTTCC |
| ANS-RA | TTTTATAGGAAAGTGACGCGGTGTG |
| ANS-FB | TGGACGGAGGGAATACTTTATACAG |
| ANS-RB | ATAATTTGAGGCTCGTTAGTGACGT |
| ACS2-FA | TGCACCCTAAATTAGTCAAATATCC |
| ACS2-RA | TGTAATGAGAACATGTCGACTAACG |
| ACS2-FB | ATCTGCGGTCCATTGTTCTCGT |
| ACS2-RB | CAAGCTAGAAGCTACACGTGAG |
| E4-FA | TCGATCTACGTCCATAAAGGAGATG |
| E4-RA | GGTTAGAGGGAAAAGTGTGGGTCAC |
| E8-FA | TTCTTAATCAGACGTATTGGGTTTC |
| E8-RA | TGAAATCTAAGAGAGAAATGGGAAC |
| E8-FB | TGAAGTCTACTAGGCGCATGTG |
| E8-RB | CTAGATATGGGTCTTTCTAGACGT |
| ERF.E1-FA | CGCCTTTCTTTGACGTACTGCTCG |
| ERF.E1-RA | GCCAAATCCTTCGAAAAATCATCTC |
| ERF.E1-FB | GTATCGGAACTAATTTCCCTTACC |
| ERF.E1-RB | TGGGCTAACATAGATATCCAAAGG |
| ERF.H1-FA | CCCTCACATAAAATGGAAGAAAGGA |
| ERF.H1-RA | TGTGTGAGGCCATGATTCACAGTT |
| ERF.H1-FB | GTGTCAATACCGCACTAGTCCT |
| ERF.H1-RB | CCGTCAGATCACTCAGTTGATATCG |
| PSY1-FA | TGGGGGTTGTCTATAATGCAGGTT |
| PSY1-RA | ACACCGCAAGAACACTGAGTGGAT |
| PSY1-FB | CCTAGTGCCAAATATAAAGGGACC |
| PSY1-RB | GAGTGATTCAGAATCCATATCCTC |
| Z-ISO-FA | GAGACTGGAAGCTATCTCAATGCA |
| Z-ISO-RA | CAACTTCTTACCACGTTCTCGGCA |
| CrtISO1-FA | CCACGTGTCAAATTCTTAAGGAGC |
| CrtISO1-RA | GGAAGTAATCTCAACGCCATTGAT |
| LCY-E-FA | TGTTGTAGCATGCAGTGGATGGAC |
| LCY-E-RA | AGTGGAAATAAGATGAGAACAAGCG |
| LCY-B-FA | GAGCATACCGAAATAGCTGAGC |
| LCY-B-RA | TTCTCCTCTTAGGTTCTGCTGC |
| LCY-B-FB | TCGCACACTATACGTAGTCTTCTC |
| LCY-B-RB | CCTTATTGATGAGAGGTTGGCTGG |
| VDE-FA | CACCAAAACCTTTAGTTGGAACTG |
| VDE-RA | ATCAGACTGATTGGTCCAAGATGC |
| VDE-FB | GTCCAAGCCCCTATGCATTCGATC |
| VDE-RB | CTGACCTCATGTAAATAGAGTGCT |
| Solyc03g114750-FA | CTCGGATGAGTGTAAGTAAGACTC |
| Solyc03g114750-RA | GGACATGACAATGACAAGCAACGT |
| Solyc03g114750-FB | GTTGATCGAGAGCTACGAGGTC |
| Solyc03g114750-RB | ACTCATGTAGCAGTTGCAGCAG |
| Solyc05g150151-FA | CCAAGAAGGACGTAGAAAATGGTG |
| Solyc05g150151-RA | GGCTAAATGGTAACCGGCTATG |
| Solyc05g150151-FB | GGATGGAGCATTAAAGATGAATCAAC |
| Solyc05g150151-RB | GTATCCCAAAACAATCACTCCTAAG |
| RIN-FA | GTTGAGGGTCATAGTTACCGACTG |
| RIN-RA | CAAATCTATGTTACCTGGAGGGCAT |
| RIN-FB | TCCTGCGTGGCGTCCTACGT |
| RIN-RB | CCTCAACTTTGGATGCGCAC |
| FUL1-FA | CAGAGAGTTATATCTACTAGGCTCG |
| FUL1-RA | TGGCTTTGAGACATATTGGCCTCT |
| FUL1-FB | CCCAGTTTATGAATCATCATGTCCG |
| FUL1-RB | GTTAGCGTGGTACCTAGTCTTGACA |
| CNR-F1 | GTTAGTGGAGTAACTACCTAGGAGT |
| CNR-R1 | GCAGAGAGAAATGTGACCATTCCTT |
| CNR-F2 | CTATGTTAGATGGCTATTACGGAGT |
| CNR-R2 | CCTCTAGCTTTGGAGGGATCAA |

**Supplementary Table 5.** **Primers used in translation efficiency assay.**

| **Primer name** | **Primer sequence (5'→3')** |
| --- | --- |
| qANS-F | AGGCCTGATTTGGCTCTTGGAG |
| qANS-R | TGGGACAAGAAGGGTGATATGGG |
| qCHI-F | AGGCTATTGTGAATGCTCCAGTTG |
| qCHI-R | TAGCACTCTCTAGCTGCACACC |
| qCHS1-F | GTCTCAGCAGCCCAAACTCTTG |
| qCHS1-R | ACGTAGGTGTCCGTCAATAGCG |
| qCHS2-F | GCTTAGTACCACAGGTGAAGGC |
| qCHS2-R | TGGAGCACAACAGTCTCAACAG |
| qF3H-F | GGCGATCACGGTCATTATTTGAGC |
| qF3H-R | TGCATCTGGTGCTGGATTCTGG |
| qF3'H-F | TGTCCGACAGGAAGAAGTCAGAAC |
| qF3'H-R | ACAGCTGCCCTAGTTTGATTGG |
| q3GT-F | ACAGGGCTTCCTTTCTTTCTTGTC |
| q3GT-R | TGTTGTTGCTGAACCCAACCTG |
| qMYB12-F | AGGCCAAGGGAAGCCTTAGTTC |
| qMYB12-R | TCACCACGTCTGGCATAATCTCC |
| qPSY1-F | ATGAGGCAGAGAAAGGCGTGAC |
| qPSY1-R | CAAGACCAAAGATGCCCATACAGG |
| qPDS-F | TTTGTGTTTGCCGCTCCAGTG |
| qPDS-R | ACAGGTACTCCGACTAACTTCTCC |
| qZISO-F | AGGATTACCAGGCATCCACAGC |
| qZISO-R | AACCTACTGAAGCTGCCACTGC |
| qZDS-F | CTCTTGGATCAAGGACATGAGGTG |
| qZDS-R | AGAACACGTGCAGTCCCATTTC |
| qCRTISO1-F | AAGGCTGAAAGATCCGAGGTAATG |
| qCRTISO1-R | TTGATGTGTCAACGGAGTTCCC |
| qCRTISO2-F | GGGAATGCCTTTCAATACCACTGC |
| qCRTISO2-R | ACACCTTGTCCTGGGAAGCAAC |
| qVDE-F | CAAAGGCTGATGCCGTTGATGC |
| qVDE-R | ACTTCGCAAGCTCTATCCTGCAC |
| qNCED1-F | ACCCAAAGCTCGACCCAGTTTC |
| qNCED1-R | ATGGCTTCTGAATCACATCGTAGC |
| qACO1-F | ACGGGAAGTACAAGAGTGTGCTG |
| qACO1-R | ACATTCGTGTCCCGTCTGTTTGTG |
| qACO5-F | CAAGGTCTCTCGAAGGAACTTTGC |
| qACO5-R | TTCGCTAGGCCAAGGTTCTCAC |
| qACS2-F | AATGTCAAGAGCCAGGGTGGTTCC |
| qACS2-R | TCCTCGCGAGCGCAATATCAAC |
| qACS4-F | TGCTCGGAGGTAGGATGGTTTC |
| qACS4-R | AATCCTTGCAAGTGCGATCTCC |
| qACS12-F | TTGGAGCTGAAAGTGGCTGTGG |
| qACS12-R | AGTTGCACCACCGGTAAGAACC |
| qETR4-F | TGTGCAGAAAGCTGGTTCAGTTG |
| qETR4-R | AGTTGAAGCCCAAGAACGACAGC |
| qEIN2-F | ATGGTTGATGGAGGAGCCCG |
| qEIN2-R | CTGCGCAAGATCTCGGTCTG |
| qE8-F | GCAACCACAGTGAAAGACCACAC |
| qE8-R | GCAGAAGTTCCATCTAGGCCTCTG |
| qDML2-F | AAGAGCGTGGAGTGTGTGAGAC |
| qDML2-R | ACGCCCTACATTTACGTCAACAGG |
| qLOXC-F | TGTGGGTGTCGATCTTTCAGGAC |
| qLOXC-R | TTTGTCCCTTTGGACCAATTGAGG |
| qPG2A-F | TGGACAAGTATGGTGGCCAAGTTC |
| qPG2A-R | TCCAGAAGGTTAAGGCCGTTGG |
| qPL1-F | GCTATTGGTGGAAGTGCTGATCCC |
| qPL1-R | TGCATCCTCGTGCTTTGTAACCTC |
| qRIN-F | TCAAAGGCATTTGCTAGGTGAGG |
| qRIN-R | TGACGTTCAAGCTGTTCCAAGTC |
| qFUL1-F | CGAAACGTCGATCTGGTTTGCTG |
| qFUL1-R | TCTCCATGCAGGAATCGTTGGC |
| qNOR-F | ACGAAGAACTCATCGTCCACTACC |
| qNOR-R | GCTCTCCGAATATTGCCTTAGCAG |
| qHY5-F | GGACAACGTCAGCTTCTGGAAGAG |
| qHY5-R | TGAGTCCCAGCTGATGGTTGAG |
| qActin-F | TCAACAACGCCTCTTTCTTCTCTC |
| qActin-R | ATCACCAGCAAATCCAGCCTTG |
